# Bxb1 and ParA recombinase-based gene assembly for multiple genes expression in plant cells

**DOI:** 10.1093/plphys/kiag690

**Published:** 2026-09-16

**Authors:** Xiangxiao Meng, Sifan Wang, Dan Huang, Huihua Wan, Yan Chen, Difa Liu, Shilin Chen, Gim Cheong Tan, Xue Cao, Wei Sun, Weiqiang Chen

**Affiliations:** State Key Laboratory for Quality Assurance and Sustainable Use of Dao-di Herbs, Institute of Chinese Materia Medica, China Academy of Chinese Medical Sciences, No. 16 South Inner Xiaojie Street, Dongzhimen, Dongcheng District, Beijing 100700, China; State Key Laboratory for Quality Assurance and Sustainable Use of Dao-di Herbs, Institute of Chinese Materia Medica, China Academy of Chinese Medical Sciences, No. 16 South Inner Xiaojie Street, Dongzhimen, Dongcheng District, Beijing 100700, China; State Key Laboratory for Quality Assurance and Sustainable Use of Dao-di Herbs, Institute of Chinese Materia Medica, China Academy of Chinese Medical Sciences, No. 16 South Inner Xiaojie Street, Dongzhimen, Dongcheng District, Beijing 100700, China; State Key Laboratory for Quality Assurance and Sustainable Use of Dao-di Herbs, Institute of Chinese Materia Medica, China Academy of Chinese Medical Sciences, No. 16 South Inner Xiaojie Street, Dongzhimen, Dongcheng District, Beijing 100700, China; Jiangmen Key Laboratory of Traditional Chinese Medicine Ingredients and Their Mechanisms of Action, Guangdong Jiangmen Chinese Medicine College, No. 4 Longwan Road, Pengjiang District, Jiangmen 529000, China; Jiangxi Qingfeng Pharmaceutical Co. Ltd., No. 65 Xingguo Road, Ganzhou 341000, China; Innovative Institute of Chinese Medicine and Pharmacy, Chengdu University of Traditional Chinese Medicine, No. 1166 Liutai Road, Wenjiang District, Chengdu 611137, China; Department of Allied Health Sciences, Faculty of Science, Universiti Tunku Abdul Rahman, Jalan Universiti, Kampar Perak 31900, Malaysia; State Key Laboratory for Quality Assurance and Sustainable Use of Dao-di Herbs, Institute of Chinese Materia Medica, China Academy of Chinese Medical Sciences, No. 16 South Inner Xiaojie Street, Dongzhimen, Dongcheng District, Beijing 100700, China; State Key Laboratory for Quality Assurance and Sustainable Use of Dao-di Herbs, Institute of Chinese Materia Medica, China Academy of Chinese Medical Sciences, No. 16 South Inner Xiaojie Street, Dongzhimen, Dongcheng District, Beijing 100700, China; State Key Laboratory for Quality Assurance and Sustainable Use of Dao-di Herbs, Institute of Chinese Materia Medica, China Academy of Chinese Medical Sciences, No. 16 South Inner Xiaojie Street, Dongzhimen, Dongcheng District, Beijing 100700, China

Dear Editor,

Delivering multiple genes into plant cells on a single DNA fragment increases the probability that all genes are expressed and co-segregated as a multigene cluster in subsequent generations of transgenic plants. Therefore, efficient and facile gene assembly methods are crucial for plant multigene engineering (Rajput et al. 2025). Currently, mainstream gene assembly technologies include type IIS restriction enzymes-based strategies (Engler et al. 2008; Weber et al. 2011; Sarrion-Perdigones et al. 2013; Pollak et al. 2019), Gibson assembly (Gibson et al. 2009), In-Fusion (Takara Bio USA, Inc.), site-specific recombinase (SSR)-based methods (Chen et al. 2006, 2010; Zhu et al. 2017; Collier et al. 2018; Zhao et al. 2025), and microbial endogenous recombination-mediated assembly (Shih et al. 2016). Among various gene assembly technologies, SSR-based methods stand out for their capability to handle large DNA sequences, high precision, and high efficiency. Those SSR-based gene assembly methods rely on in vitro site-specific recombination catalyzed by commercial enzymes (Hartley et al. 2000; Chen et al. 2006), engineered *Escherichia coli* strains expressing recombinases (Zhu et al. 2017; Zhao et al. 2025), or *Agrobacterium* strain carrying a native disarmed pRi plasmid engineered with recombination sites (Collier et al. 2018). Alternatively, we present here a Bxb1 and ParA recombinase-based gene assembly system (BPGA) that requires no special strains, purified enzymes, or complex experimental procedures. Instead, it only needs 2 rounds of heat-shock transformation of *E. coli* DH5α per assembly cycle. We validated this method by assembling 9 transcriptional units into a single vector and expressing them transiently in tobacco leaves.

BPGA comprises 1 TAC-based (Transformation-competent Artificial Chromosome-based) acceptor vector pCWA capable of delivering large DNA fragments into plants (Liu et al. 1999), along with 2 alternatively used donor vectors pCWDB and pCWDP (Fig. 1A and 1B). Bxb1 recombinase (Jusiak et al. 2019), which mediates co-integration of acceptor and donor vectors via *att* site recombination, and ParA (Gerlitz et al. 1990), which catalyzes irreversible excision of undesired DNA between directly oriented *MRS* sites within co-integration products, were pre-cloned into the donor vectors and driven by the prokaryote-active bidirectional GAL1/10 promoter from *Saccharomyces cerevisiae* (Yuan et al. 2021). Two pairs of Bxb1-recognized *att* sites were used, and cross-reactions between wild-type *att* sites (*attB* and *attP*) and mutated *att* sites (*attB^GA^* and *attP^GA^*) were not observed in vitro (Figure S1). Genes of interest were to be placed between *attB* and *attB^GA^* of pCWDB, or between *attP^GA^* and *attP* of pCWDP.

**Figure 1:**
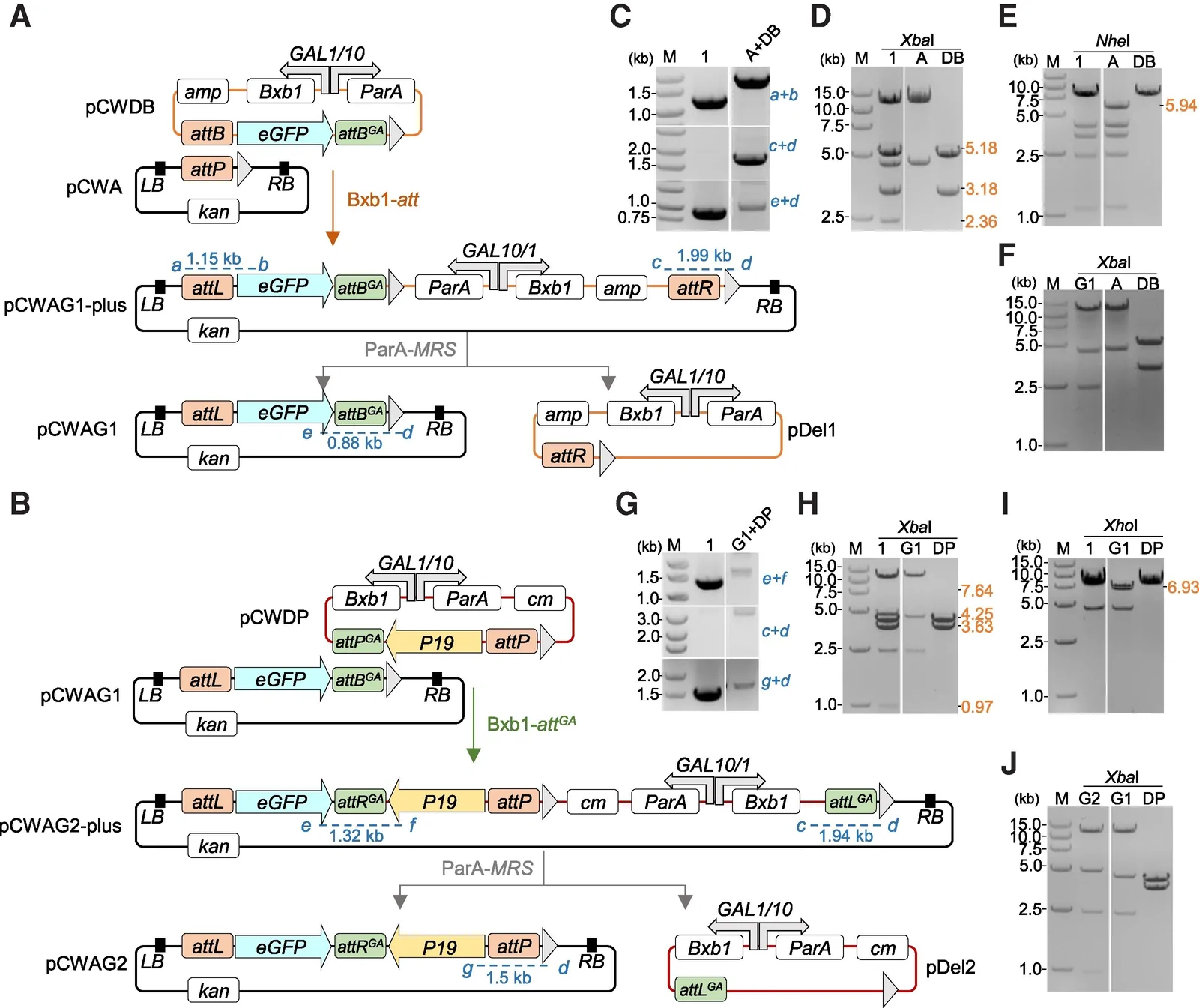
Bxb1 and ParA recombinase-based gene assembly. (A) The first round of gene assembly using pCWDB. *E. coli* co-transformed with pCWA and pCWDB would express Bxb1 that co-integrates pCWA and pCWDB through *attB* x *attP* reaction to produce pCWAG1-plus. ParA deletes unwanted DNA between 2 *MRS* sites (gray triangles) in pCWAG1-plus to yield pCWAG1 and pDel1. Primers *a* + *b* and *c* + *d* are used to amplify the co-integration junctions *attL* and *attR*, respectively; Primers *e* + *d* is used to screen for deletion products. Promoters and terminators are not shown. *amp,* ampicillin resistant gene; *kan,* kanamycin resistant gene; *GAL1/10,* bidirectional promoter from *S. cerevisiae*. *LB* and *RB*, left and right border, respectively. (B) The second round of gene assembly using pCWDP. Recombination between *attB^GA^* in pCWAG1 and *attP^GA^* in pCWDP yields construct shown as pCWAG2-plus. ParA-*MRS* recombination loops out DNA between *MRS* sites, producing pCWAG2 and pDel2. Primers *e* + *f* (*attR^GA^*) and *c* + *d* (*attL^GA^*), and *g* + *d* are used to amplify co-integrations and deletion junction, respectively. *cm,* chloramphenicol resistant gene; *P19,* suppressor of RNA silencing. (C to E) PCR, *Xba* I, and *Nhe* I analysis of representative pCWA and pCWDB co-transformed clones (lane 1). A, pCWA; DB, pCWDB; M, DNA marker in kb. (F) *Nhe* I digestion analysis of representative plasmid (lane 1) from kanamycin-resistant and ampicillin-sensitive clones transformed with plasmid DNA from clone 1 in C. (G to I) PCR, *Xba* I, and *Xho* I analysis of representative pCWAG1 and pCWDP co-transformed clones (lane 1). G1, pCWAG1; DP, pCWDP. (J) *Xba* I digestion of representative plasmids (lane 1) from kanamycin-resistant and chloramphenicol-sensitive clones transformed with plasmid DNA from clone 1 in G. Predicted restriction fragment sizes for all constructs are listed in Table S1.

Co-transformation of pCWA and pCWDB (carrying the enhanced green fluorescent protein (*eGFP*) gene) into *E. coli* was expected to generate pCWAG1-plus, pCWAG1, and pDel1 in the absence of plasmid incompatibility (Fig. 1A). However, PCR analysis of 8 kanamycin- and ampicillin-resistant clones co-transformed with pCWA and pCWDB only detected the 1.15 kb co-integration junction (primers *a* + *b*) and the 0.88 kb deletion junction (primers *e* + *d*), whereas the 1.99 kb co-integration junction (primers *c* + *d*) was absent in all 8 clones (Fig. 1C, Figure S2A–S2C), indicating that none of the clones harbored the pCWAG1-plus. *Xba* Ⅰ digestion of plasmid DNA yielded a 2.36-kb diagnostic band corresponding to pCWAG1-plus or pCWAG1, while the unique 7.30-kb band specific to pCWAG1-plus was undetectable across all 8 clones (Fig. 1D, Figure S2D). Meanwhile, the 5.18-kb and 3.18-kb bands derived from pCWDB were also observed (Fig. 1D, Figure S2D). *Nhe* Ⅰ digestion further confirmed the absence of the unique 5.94-kb band from pCWA and the 6.1-kb band from pDel1 in all tested clones (Fig. 1E, Figure S2E). Taken together, molecular characterization findings demonstrated that the clones co-transformed with pCWA and pCWDB harbored only pCWAG1 and pCWDB. Plasmid DNA from one pCWA and pCWDB co-transformed clone was re-transformed into *E. coli* and target vector pCWAG1 was recovered from kanamycin-resistant, ampicillin-sensitive clones (Fig. 1F, Figure S2F).

In the second round of gene assembly (Fig. 1B), 8 randomly selected kanamycin- and chloramphenicol-resistant clones co-transformed with pCWAG1 and pCWDP (carrying the RNA silencing suppressor *P19* from *Tomato bushy stunt virus*) yielded the 1.32 kb co-integration junction (primers *e* + *f*) and 1.50 kb deletion junction (primers *g* + *d*), whereas the 1.94 kb co-integration junction (primers *c* + *d*) was undetectable (Fig. 1G, Figure S3A–S3C). *Xba* Ⅰ digestion of plasmid DNA produced the expected 0.97-kb bands corresponding to pCWAG2-plus or pCWAG2, as well as 4.25-kb and 3.63-kb bands derived from pCWDP. By contrast, the 7.64-kb band specific to the pCWAG2-plus and the 6.5-kb band from pDel2 were absent (Fig. 1H, Figure S3D). *Xho* Ⅰ digestion further confirmed that all 8 clones lacked pCWAG1, as the diagnostic 6.93-kb band was not observed (Fig. 1I, Figure S3E). Consistent with the results obtained using pCWDB, molecular analysis verified the presence of only pCWAG2 and pCWDP in cells co-transformed with pCWAG1 and pCWDP, and the target vector pCWAG2 was recovered after a second round of transformation coupled with kanamycin-resistant, chloramphenicol-sensitive screening (Fig. 1J, Figure S3F). The net outcome from the 2 rounds of recombination was the adding of 2 genes, *eGFP* and *P19*, to the acceptor pCWA. The next round of gene addition would utilize the *attP* site of pCWAG2 along with a pCWDB derived plasmid. Hence Bxb1 mediated co-integrations alternated between the 2 sets of nonreacting *att* and *att^GA^* sites.

To exclude the possibility that the failure in detection of kanamycin-resistant acceptor vectors and co-integration vectors in co-transformed clones was due to the insufficient sampling, we analyzed an additional 95 independent co-transformed clones from each of the first 2 assembly rounds. Except for 2 clones showing PCR products from acceptor vectors, the remaining 188 clones all only detected the amplicons from target vectors (Figures S4–S11). Therefore, 98.9% (188/190) of co-transformed clones only contained a single kanamycin-resistant vector, that is, the target vector, as detected by colony PCR. Co-transformed clones on plate were pooled together for plasmid isolation, and isolated plasmid DNA was re-transformed into *E. coli*. Out of 168 tested kanamycin-resistant clones, 117 clones (69.6%) showed the ampicillin/chloramphenicol sensitivity (Figures S12 and S13). Restriction analysis on 48 ampicillin/chloramphenicol-sensitive clones revealed that all clones only contained the target vectors (Figures S14 and S15).

We hypothesize that the absence of original acceptor vectors (pCWA and pCWAG1 for the first and second round of gene assembly, respectively) and co-integration vectors (pCWAG1-plus and pCWAG2-plus in the corresponding rounds) in co-transformed clones may result from the high activity of the recombinases and the relatively low copy number of TAC vectors compared with pUC19-derived donor vectors. Under such conditions, free acceptor vectors rapidly underwent co-integration with donors and were subsequently processed into deletion products by ParA. However, the absence of pDel1 and pDel2, derived from deletion between 2 *MRS* sites within co-integration vectors, may be attributed to plasmid incompatibility, as these deletion vectors (generated at a later stage) share the same replication machinery as the donor plasmids.

Based on the above observations, the experimental workflow of the BPGA (Methods) can be greatly simplified: co-transform donor and acceptor vectors, select transformants on dual-antibiotic-supplemented plates, harvest cells directly from plates for plasmid extraction, re-transform the extracted plasmids into *E. coli*, and select on kanamycin-only plates. Randomly pick 5 to 10 kanamycin-resistant clones and screen them for ampicillin or chloramphenicol sensitivity, 69.6% clones would be ampicillin- or chloramphenicol-sensitive that contain the target vector.

Next, we used the previously characterized 7-gene andrographolide biosynthetic pathway (Wang et al. 2025) as an example to test the assembly of multiple genes following the workflow described above. Andrographolide, a diterpenoid extracted from plant *Andrographis paniculata*, has potent anti-inflammatory activities (Yang et al. 2025). At the beginning, we re-assembled *P19* into pCWAG1 to generate pCWAHT, which differs from pCWAG2 only in the orientation of the *P19* expression cassette. The 7 andrographolide biosynthetic genes, each driven by the same hyper-translatable regulatory element consisting of the CaMV 35S promoter (P35S), 5′ and 3′ untranslated regions (UTRs) from *Cowpea mosaic virus*, and the nopaline synthase terminator (Tnos), were directly amplified from pEAQ vectors (Sainsbury et al. 2009; Wang et al. 2025). These genes were inserted into pCWDB or pCWDP to replace *eGFP* or *P19*, respectively. After 7 additional rounds of gene assembly, we obtained the construct pCWHT-And7, with a total size of 41.551 kb and a T-DNA region of 25.556 kb (Fig. 2A and 2B). The stability of pCWHT-And7 in *Agrobacterium* and *E. coli* was tested by PCR or restriction digestion. Except for one *Agrobacterium* clone lost part of assembled sequences, the other 23 *Agrobacterium* clones and 24 *E. coli* clones all contained intact pCWHT-And7 after continuous long-term cultivation (Figures S16 and S17). Subsequently, *Agrobacterium* carrying pCWHT-And 7 was infiltrated into *Nicotiana benthamiana*. Liquid chromatography–mass spectrometry (LC-MS) analysis of leaf extracts collected 5 days after infiltration detected andrographolide (Fig. 2C), confirming successful expression of the andrographolide biosynthetic genes.

**Figure 2:**
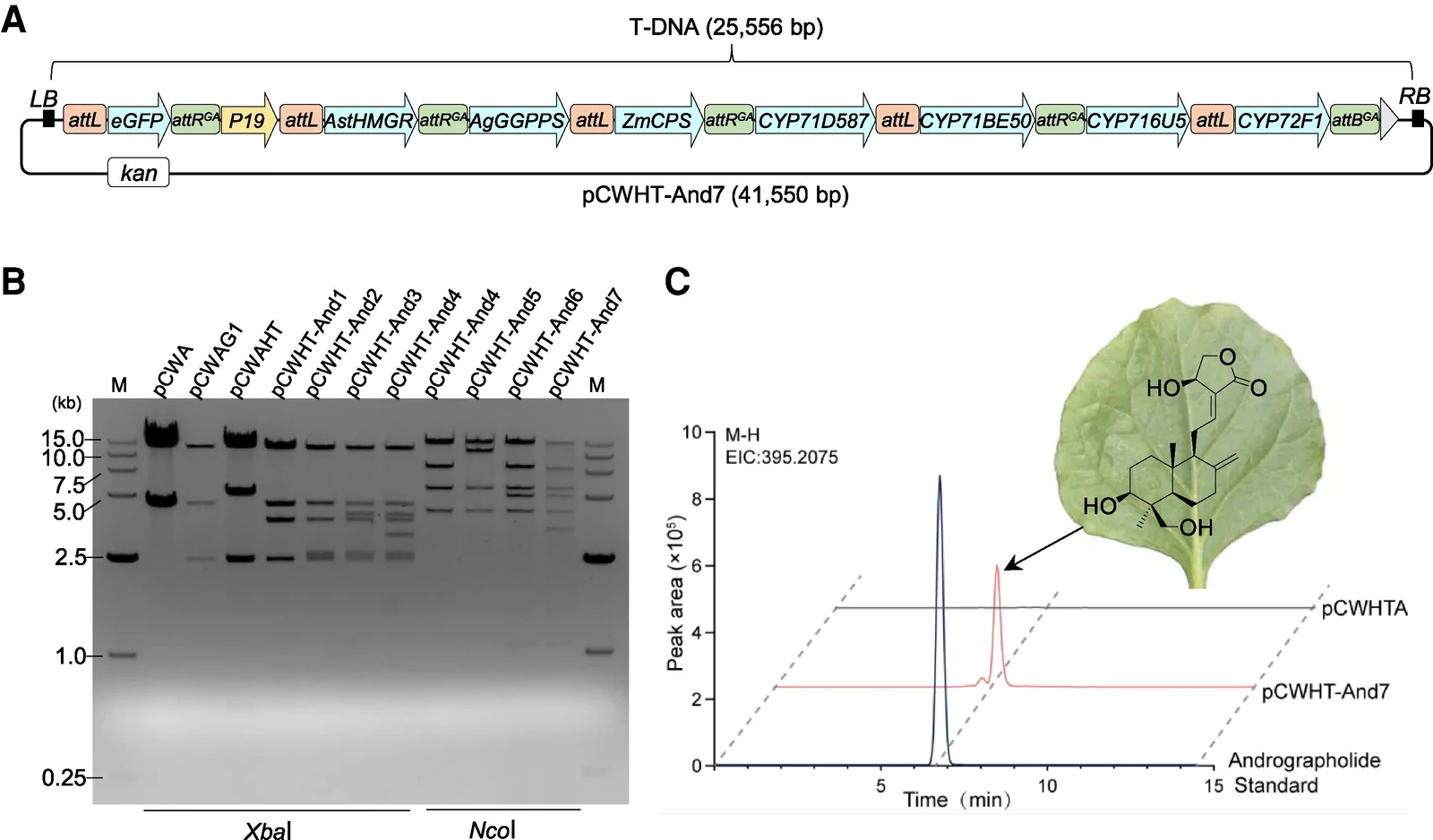
Assembly of andrographolide biosynthetic genes using BPGA and their expression in *Nicotiana benthamiana*. (A) Schematic map of pCWHT-And7 harboring a total of 9 genes, including 7 involved in andrographolide biosynthesis. All genes are transcribed left to right. Genes shaded in light blue are driven by the same hyper-translatable regulatory module composed of the CaMV 35S promoter (P35S), 5′ and 3′ UTRs from *Cowpea mosaic virus*, and the Tnos terminator; *P19* is controlled independently by P35S and CaMV 35S terminator. (B) *Xba* I and *Nco* I restriction analysis of constructs obtained at each round of gene assembly. pCWHT-And1 to And7 represent vectors generated by sequential assembly of andrographolide biosynthetic genes into pCWAHT. Predicted restriction fragment sizes are listed in Table S1. M, DNA marker in kb. (C) LC-MS profiling of metabolites extracted from *N. benthamiana* plants infiltrated with pCWHTA or pCWHT-And7.

It was worth mentioning that prior to the seventh round of gene assembly that generated pCWHT-And5, all selected clones obtained in each assembly round harbored the expected target constructs, as verified by restriction digestion or whole-plasmid sequencing. However, in the seventh round, 5 out of 8 randomly selected kanamycin-resistant and ampicillin-sensitive clones displayed aberrant restriction patterns (Figure S18). This phenomenon was also observed in the eighth and ninth assembly rounds (Figures S19 and S20). Whole-plasmid sequencing of 25 aberrant clones from the seventh (5 clones) and ninth round assembly (20 clones) revealed that these clones lacked portions of the previously assembled sequences (Figure S21). The deletions appeared to occur at distinct hotspot sites. Of 25 clones, 10 (40%) lost a fragment spanning the downstream of one poly(A) and the upstream of another poly(A), including entire or part of the second poly(A). Further, 7 out of 25 clones (28%) carried a deletion between 2 GACGAC motifs within 2 5′ UTRs, including 1 GCAGAC motif. Four clones (16%) harbored a deletion between 2 5′ UTRs, including 1 5′ UTR. Although the P35S was repeated 9 times, no sequence deletions were detected between any 2 P35S elements.

To conclude, we have developed a simple and robust gene assembly method, termed BPGA, which enables cyclic assembly of multiple genes into a single vector. Nine genes driven by the same regulatory elements were assembled and successfully expressed in *Nicotiana benthamiana* leaves. Compared with other existing gene assembly methods, BPGA requires no specialized reagents or host strains and only needs 2 rounds of *E. coli* transformation per assembly cycle. Nevertheless, sequence deletions were observed in some clones. Since 88% of the lost sequences resided between repeated sequences, this warns against the excessive reuse of identical regulatory elements in multigene construct engineering, especially the homo-polymeric tracts.

## Supplementary Material

kiag690_Supplementary_Data

## Data Availability

All data are incorporated into the article and its online supplementary material.

## References

[kiag690-B1] Chen QJ et al 2010. MISSA is a highly efficient in vivo DNA assembly method for plant multiple-gene transformation. Plant Physiol. 153:41–51. 10.1104/pp.109.152249. PMC2862421 20200068

[kiag690-B2] Chen QJ, Zhou HM, Chen J, Wang XC. 2006. A gateway-based platform for multigene plant transformation. Plant Mol Biol. 62:927–936. 10.1007/s11103-006-9065-3. 16941206

[kiag690-B3] Collier R, Thomson JG, Thilmony R. 2018. A versatile and robust *Agrobacterium*-based gene stacking system generates high-quality transgenic *Arabidopsis* plants. Plant J. 95:573–583. 10.1111/tpj.13992. 29901840

[kiag690-B4] Engler C, Kandzia R, Marillonnet S. 2008. A one pot, one step, precision cloning method with high throughput capability. PLoS One. 3:e3647. 10.1371/journal.pone.0003647. PMC2574415 18985154

[kiag690-B5] Gerlitz M, Hrabak O, Schwab H. 1990. Partitioning of broad-host-range plasmid RP4 is a complex system involving site-specific recombination. J Bacteriol. 172:6194–6203. 10.1128/jb.172.11.6194-6203.1990. PMC526800 2172207

[kiag690-B6] Gibson DG et al 2009. Enzymatic assembly of DNA molecules up to several hundred kilobases. Nat Methods. 6:343–345. 10.1038/nmeth.1318. 19363495

[kiag690-B7] Hartley JL, Temple GF, Brasch MA. 2000. DNA cloning using in vitro site-specific recombination. Genome Res. 10:1788–1795. 10.1101/gr.143000. PMC310948 11076863

[kiag690-B8] Jusiak B et al 2019. Comparison of integrases identifies Bxb1-GA mutant as the most efficient site-specific integrase system in mammalian cells. ACS Synth Biol. 8:16–24. 10.1021/acssynbio.8b00089. 30609349

[kiag690-B9] Liu YG et al 1999. Complementation of plant mutants with large genomic DNA fragments by a transformation-competent artificial chromosome vector accelerates positional cloning. Proc Natl Acad Sci U S A. 96:6535–6540. 10.1073/pnas.96.11.6535. PMC26917 10339623

[kiag690-B10] Pollak B et al 2019. Loop assembly: a simple and open system for recursive fabrication of DNA circuits. New Phytol. 222:628–640. 10.1111/nph.15625. 30521109

[kiag690-B11] Rajput R et al 2025. Multigene engineering in plants: technologies, applications, and future prospects. Biotechnol Adv. 85:108697. 10.1016/j.biotechadv.2025.108697. 40882716

[kiag690-B12] Sainsbury F, Thuenemann EC, Lomonossoff GP. 2009. pEAQ: versatile expression vectors for easy and quick transient expression of heterologous proteins in plants. Plant Biotechnol J. 7:682–693. 10.1111/j.1467-7652.2009.00434.x. 19627561

[kiag690-B13] Sarrion-Perdigones A et al 2013. GoldenBraid 2.0: a comprehensive DNA assembly framework for plant synthetic biology. Plant Physiol. 162:1618–1631. 10.1104/pp.113.217661. PMC3707536 23669743

[kiag690-B14] Shih PM et al 2016. A robust gene-stacking method utilizing yeast assembly for plant synthetic biology. Nat Commun. 7:13215. 10.1038/ncomms13215. PMC5095168 27782150

[kiag690-B15] Wang S et al 2025. Completing the biosynthesis of the clinically important diterpenoid andrographolide in *Andrographis Paniculata*. Angew Chem Int Ed Engl. 64:e202425303. 10.1002/anie.202425303. 40364767

[kiag690-B16] Weber E, Engler C, Gruetzner R, Werner S, Marillonnet S. 2011. A modular cloning system for standardized assembly of multigene constructs. PLoS One. 6:e16765. 10.1371/journal.pone.0016765. PMC3041749 21364738

[kiag690-B17] Yang S, Su P, Liu S, Wang Y. 2025. Advances of Chinese herbal medicine and its active ingredients in the treatment of antibiotic-resistant *Klebsiella pneumoniae* infections: therapeutic efficacy, mechanisms, and combination strategies. Sci Tradit Chin Med. 3:222–238. 10.1097/st9.0000000000000081.

[kiag690-B18] Yuan J, Mo Q, Fan C. 2021. New set of yeast vectors for shuttle expression in *Escherichia coli*. ACS Omega. 6:7175–7180. 10.1021/acsomega.1c00339. PMC7970545 33748631

[kiag690-B19] Zhao C, Stepanova AN, Alonso JM. 2025. DASH: a versatile and high-capacity gene stacking system for plant synthetic biology. Plant Biotechnol J. 23:3697–3712. 10.1111/pbi.70179. PMC12392928 40493021

[kiag690-B20] Zhu Q et al 2017. Development of “purple endosperm rice” by engineering anthocyanin biosynthesis in the endosperm with a high-efficiency transgene stacking system. Mol Plant. 10:918–929. 10.1016/j.molp.2017.05.008. 28666688

