# Supplementary material for: Bxb1 and ParA recombinase-based gene assembly for multiple genes expression in plant cells
Source: Plant Physiol. 2026 Sep 16;202(2):kiag690. doi: 10.1093/plphys/kiag690 (PMC13650778; doi:10.1093/plphys/kiag690)
Supplement: Supplementary file 1 [file kiag690_supplementary_data.zip › Supplementary Figures-20260806.docx]

**Supplementary Figures**

**Supplementary Fig. S1.** Cross-reactions between *att* sites and *att^GA^* sites were not observed in *in vitro* reactions. A) *att* sites used in this study. B) Linear DNA containing *attP* or *attP^GA^* was recombined with 42 bp synthetic *attB* or *attB^GA^ in vitro.* Recombination would break linear DNA into two segments of 1.3 kb and 4.2 kb. C) *in vitro* recombination products were separated on agarose gel. Lane M, DNA marker in kb. Lane 1, *attP* x *attB* without recombinase. Lane 2, 3, 4, and 5, *attP* x *attB, attP* x *attB^GA^*, *attP^GA^* x *attB*, and *attP^GA^* x *attB^GA^* with recombinase, respectively. 42 bp synthetic *attB* or a*ttB^GA^* was not visible in agarose gel. *In vitro* recombination was performed as described in (Chen and Ow, 2016).

**References**

**Chen W, Ow DW** (2016) Protocol for In Vitro Stacked Molecules Compatible with In Vivo Recombinase-Mediated Gene Stacking. Methods Mol Biol **1469:** 31-47

**Supplementary Fig. S2.** Original gel images in first round gene assembly. A), B), and C) is the upper, middle, and lower gel image of Fig. 1C, respectively. D), E), and F) corresponds to Fig. 1D, Fig. 1E, and Fig. 1F, respectively.

**Supplementary Fig. S3.** Original gel images in second round gene assembly. A), B), and C) is the upper, middle, and lower gel of Fig. 1G, respectively. D), E), and F) corresponds to Fig. 1H, Fig. 1I, and Fig. 1J, respectively.

**Supplementary Fig. S4.** PCR analysis of 95 pCWA and pCWDB co-transformed, kanamycin- and ampicillin-resistant clones with primer set *a*+*b*. The small gel on the right showed the repeated experiments of clone 17 and 21. Ninety-five clones (Lane 1-95) were analyzed. Lane M, DNA marker. Lane A+DB, PCR templated with pCWA and pCWDB mixture. One clone (Clone 17) failed to give 1.15 kb integration junction.

**Supplementary Fig. S5.** PCR analysis of 95 pCWA and pCWDB co-transformed, kanamycin- and ampicillin-resistant clones with primer set *c*+*d*. Ninety-five clones (Lane 1-95) were analyzed. Lane M, DNA marker. Lane A+DB, PCR templated with pCWA and pCWDB mixture. All clones did not yield the 1.99 kb integration junction.

**Supplementary Fig. S6.** PCR analysis of 95 pCWA and pCWDB co-transformed, kanamycin- and ampicillin-resistant clones with primer set *e*+*d*. The small gel on the right showed the repeated experiments of clone 17, 32, and 21. Ninety-five clones (Lane 1-95) were analyzed. Lane M, DNA marker. Lane A+DB, PCR templated with pCWA and pCWDB mixture. One clone (Clone 17) did not produce the 0.88 kb deletion junction.

**Supplementary Fig. S7.** PCR analysis of 95 pCWA and pCWDB co-transformed, kanamycin- and ampicillin-resistant clones with primer set *a*+*d*. The small gel on the right showed the repeated experiments of clone 57 and 79. Ninety-five clones (Lane 1-95) were analyzed. Lane M, DNA marker. Lane A+DB, PCR templated with pCWA and pCWDB mixture. Two clones (Clone 17 and 26) showed the PCR product from pCWA, the others only showed the 2.8 kb amplicon from pCWAG1.

**Supplementary Fig. S8.** PCR analysis of 95 pCWAG1 and pCWDP co-transformed, kanamycin- and chloramphenicol-resistant clones with primer set *e*+*f*. The small gel on the right showed the repeated experiments of clones 28, 40, 59, and 84. Ninety-five clones (Lane 1-95) were analyzed. Lane M, DNA marker. Lane G1+DP, PCR templated with pCWAG1 and pCWDP mixture. All clones yielded the 1.32 kb co-integration junction.

**Supplementary Fig. S9.** PCR analysis of 95 pCWAG1 and pCWDP co-transformed, kanamycin- and chloramphenicol-resistant clones with primer set *c*+*d*. The small gel on the right showed the repeated experiments of clones 12, 21, 53, and 95. Ninety-five clones (Lane 1-95) were analyzed. Lane M, DNA marker. Lane G1+DP, PCR templated with pCWAG1 and pCWDP mixture.

**Supplementary Fig. S10.** PCR analysis of 95 pCWAG1 and pCWDP co-transformed, kanamycin- and chloramphenicol-resistant clones with primer set *g*+*d*. Ninety-five clones (Lane 1-95) were analyzed. Lane M, DNA marker. Lane G1+DP, PCR templated with pCWAG1 and pCWDP mixture. All clones yielded the expected 1.5 kb co-integration junction.

**Supplementary Fig. S11.** PCR analysis of 95 pCWAG1 and pCWDP co-transformed, kanamycin- and chloramphenicol-resistant clones with primer set *e*+*d*. The small gel on the right showed the repeated experiments of clone 35. Ninety-five clones (Lane 1-95) were analyzed. Lane M, DNA marker. Lane G1+DP, PCR templated with pCWAG1 and pCWDP mixture. All clones only showed the 2.3 kb amplicon from pCWAG2.

**Supplementary Fig. S12.** Screening of pCWAG1-containing clones with kanamycin resistance and ampicillin sensitivity. pCWA and pCWDB co-transformed clones were pooled together for plasmid isolation. Isolated plasmid was re-transformed into *E. coli* and first selected on kanamycin plate. Eighty-four kanamycin-resistant clones were separately picked into 10 μL sterilized water and 2 μL was spotted on ampicillin plate, 57 clones were ampicillin-sensitive.

**Supplementary Fig. S13.** Screening of pCWAG2-containing clones with kanamycin resistance and chloramphenicol sensitivity. pCWAG1 and pCWDP co-transformed clones were pooled together for plasmid isolation. Isolated plasmid was re-transformed into *E. coli* and first selected on kanamycin plate. Eighty-four kanamycin-resistant clones were separately picked into 10 μL sterilized water and 2 μL was spotted on chloramphenicol plate, 60 clones were chloramphenicol-sensitive.

**Supplementary Fig. S14.** *Xba* I digestion analysis of kanamycin-resistant, ampicillin -sensitive clones in first round of gene assembly. Plasmid DNA from 24 ampicillin-sensitive clones (Lane 1-24) in Supplementary Fig. S12 was analyzed. Lane M, DNA marker shown in kb. Lane pCWA, pCWA was digested as a control. Predicted *Xba* I restriction patterns and stimulated agarose gel using SnapGene were shown on the right.

**Supplementary Fig. S15.** *Xba* I digestion analysis of kanamycin-resistant, chloramphenicol-sensitive clones in second round of gene assembly. Plasmid DNA from 24 chloramphenicol-sensitive clones (Lane 1-24) in Supplementary Fig. S13 was analyzed. Lane M, DNA marker shown in kb. Lane pCWAG1, pCWAG1 was digested as a control. Predicted *Xba* I restriction patterns and stimulated agarose gel using SnapGene were shown on right.

**Supplementary Fig. S16.** pCWHT-And7 stability test in *Agrobacterium* by PCR. pCWHT-And7 was introduced into *Agrobacterium* GV3101. One *Agrobacterium* clone containing intact pCWHT-And7 was cultured in liquid LB medium supplemented with kanamycin (50 μg/mL) and rifampin (50 μg/mL) for 24 h and then diluted at a volume ratio of 1:100 into fresh LB medium and incubated for another 24 h. The serial passaging procedure was repeated continuously to achieve a total culture time of 120 h. The resulting *Agrobacterium* was streaked on solid LB plate. Twenty-four *Agrobacterium* clones (Lanes 1-24) were PCR analyzed for checking the presence of *eGFP*, *P19*, *AstHMGR*, *AgGGPPS*, *ZmCPS*, *CYP71D587*, *CYP71BE50*, *CYP706U5*, and *CYP72F1*. One clone (Lane 8) failed to detect *AstHMGR*，*AgGGPPS*, *ZmCPS*, and *CYP72F1*. M, DNA marker.

**Supplementary Fig. S17.** pCWHT-And7 stability test in *E. coli* by *Nco* I digestion. Plasmid DNA from *Agrobacterium* was transformed back into *E. coli*. Twenty-four *E. coli* clones were continuously cultivated in liquid LB for 48 hours (dilute 100 times every 12 hours). Plasmids from the 24 clones were digested by *Nco* I. M, DNA marker. Predicted *Nco* I restriction patterns and stimulated agarose gel were shown on the right.

**Supplementary Fig. S18.** *Stu* Ⅰ restriction analysis of 8 kanamycin-resistant and ampicillin-sensitive clones in producing pCWHT-And5 (Left) and predicted *Stu* Ⅰ restriction pattern for pCWHT-And4 and pCWHT-And5 (Right). Five clones (black number lanes) showed the aberrant restriction pattern.

**Supplementary Fig. S19.** *Stu* Ⅰ restriction analysis of 8 kanamycin-resistant and chloramphenicol-sensitive clones in producing pCWHT-And6 (Left) and predicted *Stu* Ⅰ restriction pattern for pCWHT-And5 and pCWHT-And6 (Right). Three clones (black number lanes) showed the aberrant restriction pattern.

**Supplementary Fig. S20.** *Nco* Ⅰ restriction analysis of 8 kanamycin-resistant and ampicillin-sensitive clones in producing pCWHT-And7 (Left) and predicted *Nco* Ⅰ restriction pattern for pCWHT-And6 and pCWHT-And7 (Right). Seven clones (black number lanes) showed the unexpected restriction pattern.

**Supplementary Fig. S21.** Map of lost sequences in 25 aberrant clones. A) Sequence of 5’UTR and 3’UTR. CACGAG and poly(A) motifs are highlighted. B) Five aberrant clones in 7th round gene assembly. Structure of T-DNA from pCWHT-And5 is shown at the bottom. C) Twenty aberrant clones from 9th round gene assembly. Structure of T-DNA from pCWHT-And7 is shown at the bottom. Colored lines on the right of the numbers (Clone names) are the lost regions in the corresponding clones. Black line with arrow denotes that sequences flanking the RB outer region were also deleted.
