# Supplementary material for: Bxb1 and ParA recombinase-based gene assembly for multiple genes expression in plant cells
Source: Plant Physiol. 2026 Sep 16;202(2):kiag690. doi: 10.1093/plphys/kiag690 (PMC13650778; doi:10.1093/plphys/kiag690)
Supplement: Supplementary file 1 [file kiag690_supplementary_data.zip › Supplementary Methods-20260806.docx]

**Supplementary Methods**

***DNA constructs***

DNA between two *MRS* sites in pZH37 (Li et al., 2022) was removed by *in vitro* ParA-*MRS* reaction to yield pCW79 as described in (Chen and Ow, 2016). A synthetic *attP* from annealing of oligonucleotides *PP1F* and *PP1R* was inserted between *Spe*Ⅰ and *Pvu*ⅠⅠ of pCW79 to yield construct pCW80. pCW80 was digested with *Asc* Ⅰ and *Sca* Ⅰ, and the 683 bp fragment containing *attP*-*MRS*-*RB* was inserted between *Asc* Ⅰ and *Sca* Ⅰ of pYLTAC380H (a gift from Prof. Qinlong Zhu) to produce pCWA. *Bxb1* amplified with primer set *Bxb1-F/Bxb1-R* was inserted between *Bam*H Ⅰ and *Nhe* Ⅰ of pESC-URA to produce pESC-URA-Bxb1. Subsequently, *ParA* amplified with *BPF1/BPR1* was inserted between *Eco*R Ⅰ and *Pac* Ⅰ of pESC-URA-Bxb1 to produce pESC-URA-Bxb1-ParA. The *Bxb1* and *ParA* driven by bidirectional promoter in pESC-URA-Bxb1-ParA was amplified with primer set *BPF2/BPR2* and inserted between *Asc* Ⅰ and *Pst* Ⅰ of pCWYDB or pCWYDP to produce pCWDB-empty or pCWDP-empty, respectively. Genes with same *cis*-acting elements were directly amplified from pEAQ vectors (Wang et al., 2025) and subsequently cloned to the *Eco*R Ⅰ site of pCWDB-empty (amplified with primer set *HTFB*/ *HTRB*), or to the *Not* Ⅰ site of pCWDP-empty, (with primer set *HTFP1*/*HTRP1*). All oligonucleotides used in this study are listed in Supplementary Table S2.

***Gene assembly***

One microliter donor and acceptor vectors (100 ng for each) were introduced into DH5α competent cells (C502-02, Vazyme, Nanjing, China) via heat shock transformation. A 2%-5% aliquot of the transformed *E. coli* was plated on kanamycin (50 μg/mL) and ampicillin (100 μg/mL) supplemented Luria-Bertani (LB) plates if pCWDB derived plasmids were used, or on kanamycin (50 μg/mL) and chloramphenicol (34 μg/mL) supplemented LB plates if pCWDP derived plasmids were used. Overnight cultured *E. coli* plate was rinsed with 2 mL water to collect *E. coli* ready for plasmid isolation. One microliter isolated plasmid DNA was re-transformed into DH5α by standard heat shock transformation and 2%-5% aliquot of the transformed cells was plated on kanamycin plate. After 10-12 hours culture at 37 ℃, kanamycin-resistant colonies were picked into 10 μL sterilized water and 2 μL was spotted on ampicillin or chloramphenicol LB plate depending on which donor vectors was used. Ampicillin or chloramphenicol sensitive clones were used for analyzing plasmid structure by restriction digestion or whole plasmid sequencing. Plasmid isolation was performed using commercial FastPure Plasmid Mini Kit (DC201-01, Vazyme, Nanjing, China). For isolating constructs larger than 25 kb, 10 mL overnight culture was used.

***Tobacco leaf agroinfiltration***

DNA constructs were introduced into *Agrobacterium* GV3101 by freeze-thaw transformation and transformed cells were cultured on rifampin (50 μg/mL) and kanamycin (50 μg/mL) supplemented LB plate. Two to four clones were PCR analyzed and one positive clone was cultured 24 h in liquid LB supplemented with rifampin and kanamycin at 28 °C, 200 rpm. Cells were spun down at 5,000 rpm for 10 min and resuspended in 5 mL fresh MMA buffer (10 mM MES buffer pH 5.6; 10 mM MgCl_2_; 150 μM acetosyringone). The cell suspension was gently shaken at room temperature for 1-2 h and the OD_600_ was measured using a spectrophotometer. Cell solutions were hand infiltrated into abaxial side of the sixth tobacco leaf (counting upwards with cotyledons being leaves 1 and 2, three plants for each infiltration) using 1 mL syringe. Infiltrated plants were cultured at 18/6 light/dark for 5 days before harvesting.

***Andrographolide extraction***

Tobacco leaves were harvested 5 days post infiltration. The major vein was removed using a surgical scissor before flash frozen in liquid nitrogen. All leaves were lyophilized for 48 h, then pulverized using a PowerLyzer at 1000 rpm for 3 min. A portion of leaf powders was transferred to a new 2.0 mL screw-cap tube and 20 μL methanol was added per 1 mg leaf powder. The solutions were homogenized using PowerLyzer at 500 rpm for 5 min followed by sonication for another 10 min. The tubes were centrifuged at 13,336 *g* for 10 min, and the supernatant was transferred to a 1.5 mL tube. The solution was filtered using a 0.2 μm filter and ready for LC-MS analysis.

***LC-MS analysis***

LC-MS analysis was performed using Agilent 6546 LC-Q-TOF-MS system. Phenomenex Welch C18 column (Utimate, 3 μm, 2.1 mm × 100 mm) was for chromatographic separation with a gradient elution of 0.1% formic acid in water (mobile phase A) and 100% acetonitrile (mobile phase B). Gradient profile: 0-2 min, 5% B; 2.5 min, 18.5% B; 10.5 min, 41% B; 11 min, 59% B; 12-16 min, 100% B. Injection volume: 4 μL; Flow rate: 0.3 mL min^-1^; column temperature: maintained at 40 °C. The ESI source parameters were as follows: discharge current 6 mA, capillary temperature 350 ℃, heater temperature 350 ℃, Data-dependent acquisition was used for data acquisition under negative ion mode. The full scan and fragment spectra were collected with resolutions of 100 and 1,500, respectively. Mass spectrometric detection was performed in negative ion mode, and data acquisition and processing were carried out using MassHunter software (Agilent Technologies, Santa Clara, CA, USA).

**References**

**Chen W, Ow DW** (2016) Protocol for In Vitro Stacked Molecules Compatible with In Vivo Recombinase-Mediated Gene Stacking. Methods Mol Biol **1469:** 31-47

**Li R, Han Z, Yin Q, Li M, Zhang M, Li Z, Wang P, Jiang L, Ow DW** (2022) Target Lines for in Planta Gene Stacking in Japonica Rice. Int J Mol Sci *23*, 9385. https://doi.org/10.3390/ijms23169385

**Wang S, Liang M, Chen W, Wan H, Meng X, Zhu X, Lu Y, Shen Q, Jiang C, Xie N, Chen S, Jia M, Sun W** (2025) Completing the Biosynthesis of the Clinically Important Diterpenoid Andrographolide in Andrographis Paniculata. Angew Chem Int Ed Engl **64:** e202425303
