# Supplementary material for: Bxb1 and ParA recombinase-based gene assembly for multiple genes expression in plant cells
Source: Plant Physiol. 2026 Sep 16;202(2):kiag690. doi: 10.1093/plphys/kiag690 (PMC13650778; doi:10.1093/plphys/kiag690)
Supplement: Supplementary file 1 [file kiag690_supplementary_data.zip › Supplementary Tables-20260806.docx]

**Supplementary Tables**

| **Supplementary Table S1.** Predicted restriction patterns for constructs used in the article | | | |
| --- | --- | --- | --- |
| Constructs | Restriction enzyme | Predicted restriction fragments in bp | Corresponding figures |
| pCWA | *Xba* I | 12,148; 4,487 | Fig. 1D, Fig. 1F |
| pCWDB |  | 5,181; 3,181 |  |
| pCWAG1-plus |  | 12,148; 7,307; 3,181; 2,361 |  |
| pCWAG1 |  | 12,149; 4,364; 2,361 |  |
| pDel 1 |  | 6,124 |  |
| pCWA | *Nhe* I | 5,938; 3,931; 3,261; 2,379; 1,126 | Fig. 1E |
| pCWDB |  | 8,362 |  |
| pCWAG1-plus |  | 7,669; 6,631; 3,931; 3,261; 2,379; 1,126 |  |
| pCWAG1 |  | 8,176; 3,931; 3,262; 2,379; 1,126 |  |
| pDel 1 |  | 6,124 |  |
| pCWAG1 | *Xba* I | 12,149; 4,364; 2,361 | Fig. 1H, Fig. 1J |
| pCWDP |  | 4,253; 3,625 |  |
| pCWAG2-plus |  | 12,149; 7,643; 3,625; 2,361; 974 |  |
| pCWAG2 |  | 12,149; 4,808; 2,361; 974 |  |
| pDel 2 |  | 6460 |  |
| pCWAG1 | *Xho* I | 7,563; 6,926; 4,385 | Fig. 1M |
| pCWDP |  | 7,878 |  |
| pCWAG2-plus |  | 10,416; 7,563; 4,388;4,385 |  |
| pCWAG2 |  | 8344; 7,563; 4,385 |  |
| pDel 2 |  | 6460 |  |
| pCWA | *Xba* I | 12,148; 4,487 | Fig. 2B |
| pCWAG1 |  | 12,149; 4,364; 2,361 |  |
| pCWAHT |  | 12,149; 4,487 |  |
| pCWHT-And1 |  | 12,149; 4,364; 3,588; 2,359; 603 |  |
| pCWHT-And2 |  | 12,149; 4,383; 3,588; 2,488; 2,359; 603 |  |
| pCWHT-And3 |  | 12,149; 4,364, 3,831, 3,588, 2,488, 2,359, 603 |  |
| pCWHT-And4 |  | 12,148; 4,383; 3,831; 3,588; 3,036; 2,488; 2,359; 603 |  |
| pCWHT-And4 | *Nco* I | 14,367; 8,061; 5,623; 4,043; 291; 51 |  |
| pCWHT-And5 |  | 14,367; 11,057; 5,616; 4,041; 291; 51 |  |
| pCWHT-And6 |  | 14,367; 8,033; 5,623; 5,026; 4,043; 1,063; 291; 51 |  |
| pCWHT-And7 |  | 14,367; 7,819; 5,623; 5,026; 4,043; 3,267; 1,063; 291; 51 |  |

| **Supplementary Table S2**. oligonucleotides used in this study | |
| --- | --- |
| Name (alternative name) | sequence (5'-3') |
| PP1F | gggtttgtaccgtacaccactgagaccgcggtggttgaccagacaaaccactga |
| PP1R | ctagtcagtggtttgtctggtcaaccaccgcggtctcagtggtgtacggtacaaaccc |
| Bxb1-F | gtcaaggagaaaaaaccccgATGAGAGCCCTGGTAGTCA |
| Bxb1-R | tcggttagagcggatcttagCTACGACATCCCGGTGTGT |
| BPF1 | TAAGAATTTTTGAAAATTCGatggcgacgcgagagca |
| BPR1 | TCTGGCGAAGAATTGTTAATctaagcccttttattttgctgctgc |
| BPF2 | acgcccacatatgagcccggGAGCGACCTCATGCTATACCTGA |
| BPR2 | aattacggctcctcgctgcaCTTCGAGCGTCCCAAAACCTTCT |
| a (LB-R) | TGGCTGGTGGCAGGATATATT |
| b (eGFP-5N) | cgtttacgtcgccgtcc |
| c (Int1-R) | gggaaccggagctgaatgaa |
| d (BPR5) | AAGGCGGGAAACGACAATCT |
| e (BPF5) | AGCTCCTGTTTAGCAGGTCG |
| f (P19F) | ATGGAACGAGCTATACAAGGAAAC |
| g (P19R) | TTACTCGCTTTCTTTTTCGAAGG |
| HTFB | caagccgggaattggatccgTGTAAAACGACGGCCAGTG |
| HTRB | tgcggccgcatacggtaccgTGACCATGATTACGCCAAGC |
| HTFP1 | tggatcggatccgaattcgcTGTAAAACGACGGCCAGTG |
| HTRP1 | atcgttgacgactcggctacgcggccgcTGACCATGATTACGCCAAGC |
